# Predictive model for aminoglycoside induced ototoxicity

**DOI:** 10.3389/fneur.2024.1461823

**Published:** 2024-11-01

**Authors:** Adebolajo A. Adeyemo, Josephine Adeolu, Joshua O. Akinyemi, Olayemi O. Omotade, Odunayo M. Oluwatosin

**Affiliations:** ^1^Institute of Child Health, College of Medicine, University of Ibadan, Ibadan, Nigeria; ^2^Department of Otolaryngology, University College Hospital, Ibadan, Nigeria; ^3^Department of Epidemiology and Medical Statistics, College of Medicine, University of Ibadan, Ibadan, Nigeria; ^4^Department of Surgery, College of Medicine, University of Ibadan, Ibadan, Nigeria

**Keywords:** aminoglycosides, ototoxicity, predictive model, model validation, tuberculosis

## Abstract

**Background:**

Irreversible hearing loss is a well-known adverse effect of aminoglycosides, however, inability to accurately predict ototoxicity is a major limitation in clinical care. We addressed this limitation by developing a prediction model for aminoglycoside ototoxicity applicable to the general population.

**Methods:**

We employed a prospective non-drug-resistant tuberculosis (TB), non-HIV/AIDS cohort of 153 adults on Streptomycin based anti-TB therapy. High frequency pure-tone audiometry was done at regular intervals throughout the study. Clinical and audiological predictors of ototoxicity were collated and ototoxic threshold shift from the baseline audiogram computed. The prediction model was developed with logistic regression method by examining multiple predictors of ototoxicity. Series of models were fitted sequentially; the best model was identified using Akaike Information Criterion and likelihood ratio test. Key variables in the final model were used to develop a logit model for ototoxicity prediction.

**Results:**

Ototoxicity occurred in 35% of participants. Age, gender, weight, cumulative Streptomycin dosage, social class, baseline pure tone average (PTA) and prior hearing symptoms were explored as predictors. Multiple logistic regression showed that models with age, cumulative dosage and baseline PTA were best for predicting ototoxicity. Regression parameters for ototoxicity prediction showed that yearly age increment raised ototoxicity risk by 5% (AOR = 1.05; CI, 1.01–1.09), and a gram increase in cumulative dosage increased ototoxicity risk by 7% (AOR = 1.05; CI, 1.05–1.12) while a unit change in baseline log (PTA) was associated 254% higher risk of ototoxicity (AOR = 3.54, CI: 1.25, 10.01). Training and validation models had area under the receiver operating characteristic curve as 0.84 (CI, 0.76–0.92) and 0.79 (CI, 0.62–0.96) respectively, showing the model has discriminatory ability.

**Conclusion:**

This model can predict aminoglycoside ototoxicity in the general population.

## 1 Introduction

Aminoglycoside-induced ototoxicity is a complex process which has not been fully elucidated. The irreversible drug-induced ototoxicity is a dose-dependent adverse effect (1) that is exacerbated by both the wide variation in the drug pharmacokinetics in humans, as well as the restricted therapeutic bounds of aminoglycosides (2). The cochlear neuro-epithelium is the primary lesion site in aminoglycoside ototoxicity (3), with sequential destruction of the outer hair cells, starting at the basal turn of the cochlear before spreading to the upper cochlear turns and the inner hair cells (4), followed by damage to the nerve fibres and ganglion cells.

Aminoglycosides possess a highly polar, polycationic structure which prevents adequate absorption following oral ingestion, however parenteral administration leads to a complete absorption (5). The highly polar nature also facilitates attraction between the positively charged drug and the negatively charged glycocalyx on the apical surface of the inner ear sensory hair cells; a competitive binding between aminoglycosides and Ca^2+^ ions to the stereocilia occurs, causing a reversible interference with transduction channels (3).

There is rapid uptake of aminoglycosides into the inner ear with accumulation of the drug in the endolymph and perilymph of the inner ear within minutes of parenteral administration (6). The drug persists in the inner ear long after clearance from the bloodstream and this is probably responsible for the deferred inner ear sensory cell death seen after completion of the medical therapy (7). Despite the rapid accumulation of aminoglycosides in the inner ear, cell death is not immediate rather sensory cell deaths begins after a period of several days (8, 9).

Inhibition of protein synthesis (10) and production of Reactive Oxygen Species (ROS) by aminoglycosides are possible death mechanisms for the inner ear sensory cells (11). Moreover, the outer hair cells have greater sensitivity to free radicals’ injury than other cell types within the cochlea. While the outer hair cells in the cochlea base have greater sensitivity to injury than cells at the apices of the cochlea spiral (12). This pattern of sensitivity to injury mirrors the progressive base to apex gradient of hair cell destruction and the preferential outer hair cell damage seen in aminoglycoside exposure (7).

The clinical usage of aminoglycosides took off with the discovery of Streptomycin by Waksman in 1944 (7), this milestone, along with the use of other potent antibiotics heralded an effective anti-TB drug campaign. A preliminary success was achieved (13), but TB eradication efforts were not fully successful, in recent times the prevalence of the disease has increased. A third of the global population is already infected with *Mycobacterium tuberculosis* and are liable to succumb to a full-blown disease (2, 14). Notwithstanding the ototoxic side effect, aminoglycosides such as Streptomycin, Kanamycin, and Amikacin are still key pillars in the anti-TB drug profile (15).

Apart from the deployment in the anti-TB crusade, aminoglycosides are massively prescribed globally but more so in many developing countries (16). Aminoglycosides are relatively cheap but powerful drugs; thus, they are widely employed in the treatment of varied infections, this probably accounts for the huge consumption in low-and medium-income countries, and also a significant contributor to the huge burden of hearing loss in these countries (17). However, irrespective of the clime, clinicians often face a conundrum when discussing with patients and/or relations the risks of developing ototoxicity following a treatment regime with aminoglycosides. The inability to accurately predict the risk of ototoxicity could impair decision to institute and/or accept prescribed treatment. Thus, development of a predictive model for ototoxicity after exposure to aminoglycosides will be an effective tool to aid physician and patient communication and guide clinical practize. Such a tool can complement serum monitoring of aminoglycosides during therapy, and if serum monitoring is unavailable the tool may be a crucial aid in clinical decision making. Therefore, we sought to develop a predictive model for aminoglycoside ototoxicity applicable to the general population.

## 2 Materials and methods

### 2.1 Study design

This was a prospective study among a cohort of individuals diagnosed with pulmonary TB undergoing treatment with streptomycin based anti-TB drugs (18). The participants were recruited successively as diagnosis was made and informed consent provided from multiple Directly Observed Therapy-Short course (DOTS) centres in Ibadan, southwest Nigeria. Ibadan is a cosmopolitan city and the largest metropolitan area in Nigeria by geographical area.

### 2.2 Study population

The study participants were physician diagnosed TB patients (≥18 years) undergoing second line of treatment with anti-TB drugs which included Streptomycin. These were patients who had a prior diagnosis of Tb following positive sputum smear microscopy, i.e., identification of tuberculous bacilli in sputum samples. These patients had previously treatment for TB and declared cured or completed a full course of treatment and once again developed sputum smear-positive TB or a sputum smear positive patient who while on treatment remains or became smear positive again 5 months or more after commencement of treatment or a TB patient who completed at least 4 weeks of category I treatment and returned smear positive after at least 8 weeks of interruption of treatment. The participants must also have normal hearing thresholds as assessed by pure tone audiometry at baseline. That is before the second line of treatment with anti-TB drugs which included Streptomycin was commenced. Those with hearing loss secondary to any pathology as assessed by baseline audiometry, and/or signs of active ear infection, and/or those with concurrent HIV/AIDS infection, and/or those using additional medications with ototoxic potentials and/or nephrotoxic potentials were excluded from the study.

### 2.3 Measures

All relevant socio-demographic (including but not limited to age, gender, educational attainment, socio-economic status) and medical information were documented with the aid of a modified questionnaire (19). Baseline audiometry (between 125 Hz and 8,000 Hz) was done with a KUDU wave audiometer (Geoaxon, Pretoria, South Africa) for every participant, subsequently the audiometry was repeated weekly throughout the first 2 months of the therapy also known as the intensive phase. After that the audiometry (between 125 Hz and 16,000 Hz) was repeated monthly for the following 6 months of the therapy for all the participants. The pure tone average (PTA) were calculated from the audiograms of all the subjects (20) and these were examined. The American Speech-Language-Hearing Association guidelines were utilized in computing ototoxic threshold shift from the baseline audiogram (21).

Serum streptomycin levels was not measured during the course of therapy (15). The guidelines of the Nigerian National Tuberculosis and Leprosy Control Programme mandates single intramuscular Streptomycin injection every day for patients undergoing TB re-treatment thus single daily dosing regimen was adopted for this study. All the drugs used in TB management are supplied centrally by the National Tuberculosis and Leprosy Control Programme.

### 2.4 Statistical model development and validation

The dataset was partitioned into two in a ratio of 70%: 30% for training and validation datasets, respectively. We used logistic regression method to develop the prediction model by examining multiple predictors of ototoxicity: age, gender, weight, cumulative dosage of aminoglycoside, social class, baseline PTA (20) and prior ear/hearing related symptoms such as prior hearing loss, tinnitus and vertigo.

A series of models were fitted sequentially, and the best model was identified based on Akaike Information Criterion and likelihood ratio test. Key variables in the final Logit model was then used to develop a logit model for prediction of ototoxicity. The final prediction model chosen in the training dataset maximized discrimination [i.e., the area under receiver operating characteristic (ROC) curve (AUC)] without poor calibration (i.e., *p* < 0 0.05 on the Hosmer-Lemeshow *χ*2 goodness-of-fit test). The chosen model was applied to the validation dataset and the predictive accuracy of the model measured as the AUC (for discrimination) and Hosmer-Lemeshow χ2 goodness-of-fit statistic (for calibration) reported. The statistical tests were done at a 2-sided significance level of 0.05.

### 2.5 Ethical considerations

Ethical approval was received from the Oyo State Ministry of Health, Ethical Review Committee (AD 13/479).

## 3 Results

### 3.1 Study population

One hundred and fifty-three adults completed the study; these individuals completed their anti-TB drug regimen and had regular audiometry assessment throughout the study period. The study population is made up 95 males and 58 females. The ages of the participants were classified into three age groups: <40 years, 40–49 years and ≥ 50 years. Those in the <40 years age group made up 44% of the study population followed by the 40–49 years age (32%). The mean age of participants was 41.4 (SD = 12.7) years. Most of the study participants (77.7%), had 9 years or less of accumulated formal education, while only 9.2% had tertiary education. The educational attainment profile was mirrored in the occupation pattern; 73.6% were either laborers, petty traders or unemployed. Grouping of the participants into three weight classes was done: <50 kg (32%), 50–59 kg (39.2%) and > 60 kg (28.1%) respectively. Based on the weight classification, 63.4% individuals received 0.75-gram dosage of Streptomycin while 32% received 1gram dosage of Streptomycin daily for 2 months. The mean cumulative dosage was 50.8 grams (SD = 13.9). We applied natural logarithm to transform the baseline PTA and the mean was 3.28 (SD = 0.47).

The ASHA guidelines used in the assessment of the audiograms of the study population showed that approximately 35% of the study population developed ototoxicity by the end of the study period.

### 3.2 Development and validation of prediction model

The results of the Logistic regression of Age, Gender, Weight, Cumulative dose, Social Class, Baseline PTA and Pre-existing ear symptoms is shown in Table 1. Age (as a continuous variable), Cumulative dose (as a continuous variable) and Log of baseline PTA were the only variables with significant *p*-values. (Table 1).

**Table 1:** Results of logistic regression with independent variables.

Variables		Coefficients	Odds ratio	Lower confidence interval	Upper confidence interval
Age		0.05177	1.05	1.01	1.10*
Gender	Female		1		
	Male	0.29309	1.34	0.51	3.52
Weight (kg)	<50		1		
	50–59	−0.621183	0.54	0.19	1.55
	≥60	−0.724923	0.48	0.16	1.47
Cumulative dosage		0.08224	1.09	1.05	1.13*
Social class	Very low		1		
	Low	−0.804919	0.45	0.09	2.26
	High	−0.214487	0.81	0.19	3.38
	Very high	0.1579889	1.17	0.18	7.58
Previous ear symptoms	No		1		
	Yes	0.105629	1.11	0.43	2.90
Baseline audiogram (Log PTA)		1.365582	3.92	1.24	12.40*

* *p* < 0.05.

The model incorporating all independent variables was compared with the model incorporating only age and cumulative dosage as independent variables as well as age, cumulative dosage and Log of baseline PTA (see Table 2). The model incorporating only age, cumulative dosage and Log of baseline PTA as independent variables had better AIC.

**Table 2:** Comparison of models for predicting ototoxicity.

Model	AIC	likelihood ratio test (LRT)	*p* value for LRT	Goodness of fit *p* value
Ototoxicity against all variables	159.5805	35.6	*p* < 0.001	p < 0.001
Ototoxicity with age and cumulative dose	157.3782	27.17	p < 0.001	p < 0.001
Ototoxicity with age, cumulative dose, and Baseline audiogram (Log PTA)	153.0145	33.54	p < 0.001	p < 0.001

The regression parameters for prediction of ototoxicity are shown in Table 3. This revealed that as the age increases by a year the risk of ototoxicity increases by 5%, and as the cumulative dosage of Streptomycin increases by 1gram the probability of ototoxicity increases by 7%. Similarly, a unit increase in the Log of baseline PTA was associated with 254% higher risk of ototoxicity.

**Table 3:** Regression parameters for predicting ototoxicity.

Oto (yes/no)	Coefficient	OR (95% CI)	*p*-value
Age	0.0462154	1.05 (1.01, 1.09)	0.012
Cumulative dosage	0.077249	1.08 (1.05, 1.12)	0.000
Baseline audiogram (Log PTA)	1.263387	3.54 (1.25, 10.01)	0.017

**Figure 1:**
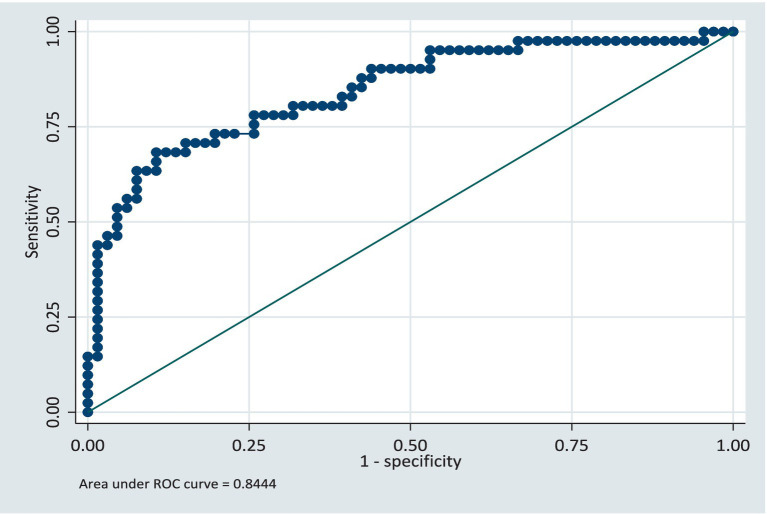
Receiver operating characteristics (ROC) curve for the training dataset.

**Figure 2:**
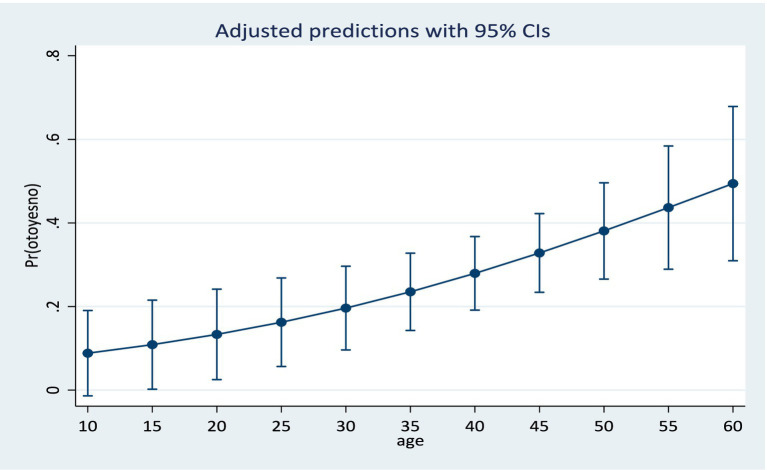
Predicted probabilities for age at means of cumulative dosage and log baseline audiogram.

**Figure 3:**
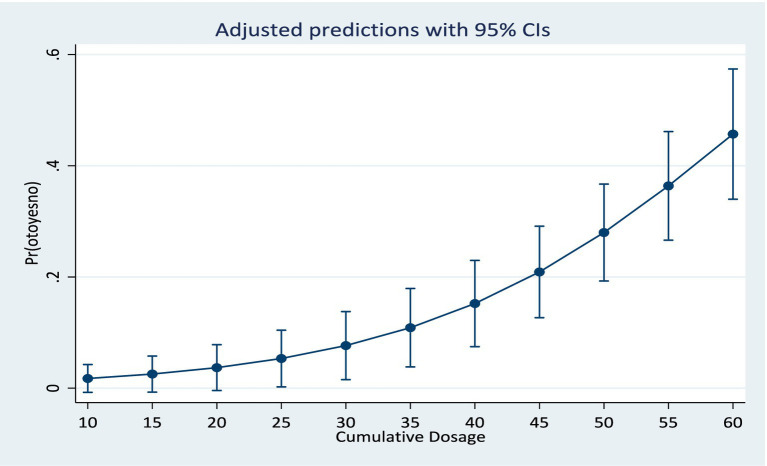
Predicted probabilities for cumulative dosage at means of age and log baseline audiogram.

Results from the training model showed that the area under the ROC curve (Figure 1) was 0.84 (CI: 0.76, 0.92). Sensitivity values and specificity values at various cut-off points are presented in the Supplementary material. The predicted probabilities for different values of age and cumulative dosage are shown in Figures 2, 3 respectively.

**Figure 4:**
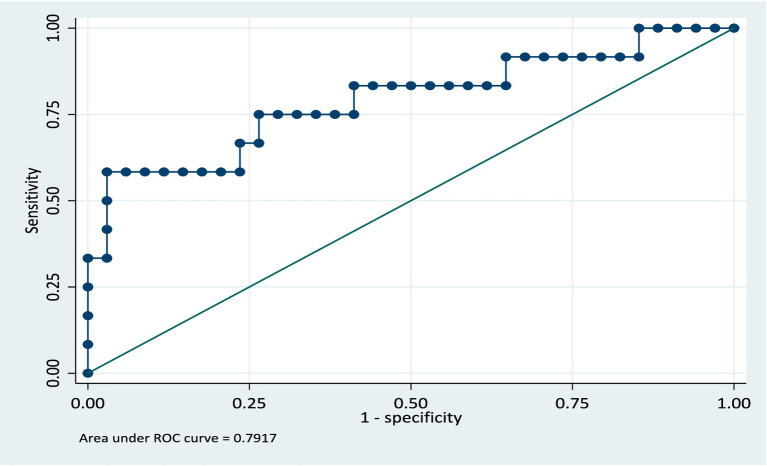
ROC curve for validation model.

In the validation dataset, the area under the ROC curve was 0.79 (CI: 0.62, 0.96) see Figure 4. This shows that the model still retained its discriminatory ability even though the area the ROC curve is slightly reduced. The slight reduction of the area under the ROC curve being likely due to the sample size, however the confidence intervals for both the training and validation models are similar. The model was further validated on the full dataset and the AUC was 0.82 (CI: 0.75, 0.90). The model retained its performance when applied to the same population of participants. We anticipate that if this model is applied to a group of patients similar in characteristics to the study population, there will be a 5% reduction in the AUC, specifically, AUC = 0.77.

A predictive model that estimates the probability/risk of ototoxicity based on the regression parameters from the training model is shown below:


$$p=\frac{\exp\left[\begin{array}{l} -12.18594+\mathrm{age}*0.0606671+\\ \mathrm{Cumulative}\;\mathrm{Dosage}*0.0727272+Log_{PTA}*1.6255 \end{array}\right]}{1+\exp\left[\begin{array}{l} -12.18594+\mathrm{age}*0.0606671+\\ \mathrm{Cumulative}\;\mathrm{Dosage}*0.0727272+Log_{PTA}*1.6255 \end{array}\right]}$$

## 4 Discussion

Accurate prediction of ototoxicity before commencement of a drug regimen with aminoglycosides is important to guide patient counseling sessions and enable patient and relations to make informed consent about treatment plans. Risk assessment of hearing loss could also help determine the patients who will benefit from more vigorous monitoring during the treatment regimen. Considering the huge negative impact of hearing loss on the quality of life developing a prediction model that can provide risk assessment of ototoxicity is very desirable.

A simple prediction model was developed for predicting ototoxicity within eight (8) months following onset of aminoglycoside therapy. The model is based on clinical factors such as the audiometry tests, cumulative drug dosage as well as pre-treatment variables such as age, weight, and pre-existing ear/hearing lesions. The model confirmed that age, baseline PTA and cumulative dosage were the three most important predictors of hearing loss in a patient on aminoglycoside therapy. The model is applicable to applicable to patient population with characteristics similar to the study population. These include individuals receiving a wide range of cumulative dosage of aminoglycosides, from 20 g to 90 g. This wide range of cumulative dosage covered in the model is beneficial, since aminoglycoside dosage is determined by body weight, it suggests that the model will cover the cumulative dosage of the prescription of most individuals.

The treatment duration covered within the model (8 months) affords a significant period of time after completion of therapy. This considerable time span helps to accommodate even the delayed elimination of aminoglycoside from the inner ear. This makes the model suitable for most clinical scenarios warranting aminoglycoside therapy. Moreover, this prediction model may reflect the true hearing outcomes in patients on aminoglycoside therapy considering the long duration period after completion of aminoglycoside therapy. The model is also particularly suited for prediction of ultra-high frequency hearing loss because ultra-high frequency (up to 16,000 Hz) audiometry was done in all the study subjects. This is clinically important because ultra-high frequency loss denotes early-onset ototoxicity in individuals on aminoglycoside therapy, this early-onset ototoxicity may not manifest with immediate functional impairment of hearing as the speech frequencies are spared at this early stage. However, progression of the hearing loss in the absence of counter measures will lead to involvement of the speech frequencies, noticeable hearing impairment and attendant impacts on the quality of life.

The model showed the suitability of routine clinical data in predicting aminoglycoside ototoxicity in patients with reasonable accuracy. The statistical model we described is based solely on clinical factors, even though genetic susceptibility has been described in aminoglycoside ototoxicity (22), the lack of inclusion of genetic factors may not significantly affect the model. It has been previously shown that the incorporation of genetic susceptibility factors into statistical models previously built on only clinical factors, may not alter significantly the predictive ability of the model (23). Several studies have shown that genetic susceptibility factors do not provide prediction models that are as good as models based on clinical factors (24–26), though combining genetic factors to clinical factors could produce marginal increase in the predictive ability of the models (24). Thus, our model may be applicable for practical usage without contemplating the genetic susceptibility of the subjects. The readily available clinical data is adequate for prediction.

A distinctive advantage we believe this model have is the applicability to the general population. Some other predictive models that were developed previously used specialized patient groups that included drug resistant TB patients and/or HIV/AIDS patients (27), since these patient groups are not generalizable to the rest of the population there is an obvious limitation to the use of the predictive model. We aimed to bypass that limitation by using a non-drug resistant TB, non-HIVAIDS patient cohort in the model development. The similarity of our patient cohort to the general population implies that our model is relevant for use in people who are receiving aminoglycosides for any infective cause. However, it is still necessary to subject this model to external validation by evaluating how the model will perform in a separate patient population. This is a limitation of this study and a focus for future research.

The ultimate objective of the statistical model is to provide support to physicians in clinical decision making. The model is positioned to be a useful resource tool when counseling patients on the possibility of ototoxicity occurrence following a course of aminoglycoside therapy care, this will enable the patient and/or relations make informed decision on the planned course of action. The prediction of ototoxicity may also guide clinical decision-making process, including consideration of alternative therapies. These clinical decisions can be derived without the need for additional laboratory tests or separate clinical evaluations, thus the statistical model empowers both the clinician and the patient, especially those in resource constrained environments to take informed decisions and plan additional protective steps to mitigate ototoxicity. This model can be can easily incorporated into a mobile app that requires only the input of the age of the patient, the expected cumulative dosage of aminoglycoside, the baseline PTA and the probability of aminoglycoside-induced ototoxicity can be readily forecasted from the app.

## 5 Conclusion

The predictive model showed that the age of an individual, the cumulative dosage of aminoglycoside and baseline PTA are sufficient to predict the probability of subsequent occurrence of ototoxicity. The model can improve treatment plans by enabling informed decision making by the clinician and the patient. The readily available clinical data incorporated in the model implies that it can be used easily both in high—and low-resource settings. More importantly the model is not restricted to specialized patient groups, rather it is suitable to the general population.

## Data Availability

The original contributions presented in the study are included in the article/Supplementary material, further inquiries can be directed to the corresponding authors.
