# Supplementary material for: Predictive model for aminoglycoside induced ototoxicity
Source: Front Neurol. 2024 Nov 1;15:1461823. doi: 10.3389/fneur.2024.1461823 (PMC11563990; doi:10.3389/fneur.2024.1461823)
Supplement: Supplementary file 1 [file Data_Sheet_1.pdf]

Detailed report of sensitivity and specificity

Cutpoint	Sensitivity	Specificity	Correctly classified	LR+	LR-
(>= .0052..)	100.00%	0.00%	38.32%	1.0000	
(>= .0082..)	100.00%	1.52%	39.25%	1.0154	0.0000
(>= .0126..)	100.00%	3.03%	40.19%	1.0312	0.0000
(>= .0135..)	100.00%	4.55%	41.12%	1.0476	0.0000
(>= .0179..)	97.56%	4.55%	40.19%	1.0221	0.5366
(>= .0210..)	97.56%	6.06%	41.12%	1.0386	0.4024
(>= .0251..)	97.56%	7.58%	42.06%	1.0556	0.3220
(>= .0394..)	97.56%	9.09%	42.99%	1.0732	0.2683
(>= .0516..)	97.56%	10.61%	43.93%	1.0914	0.2300
(>= .0578..)	97.56%	12.12%	44.86%	1.1102	0.2012
(>= .0619..)	97.56%	13.64%	45.79%	1.1297	0.1789
(>= .0626..)	97.56%	15.15%	46.73%	1.1498	0.1610
(>= .0685..)	97.56%	16.67%	47.66%	1.1707	0.1463
(>= .0687..)	97.56%	18.18%	48.60%	1.1924	0.1341
(>= .0756..)	97.56%	19.70%	49.53%	1.2149	0.1238
(>= .0813..)	97.56%	21.21%	50.47%	1.2383	0.1150
(>= .0846..)	97.56%	22.73%	51.40%	1.2626	0.1073
(>= .0856..)	97.56%	24.24%	52.34%	1.2878	0.1006
(>= .0903..)	97.56%	25.76%	53.27%	1.3141	0.0947
(>= .0908..)	97.56%	27.27%	54.21%	1.3415	0.0894
(>= .0961..)	97.56%	28.79%	55.14%	1.3700	0.0847
(>= .0997..)	97.56%	30.30%	56.07%	1.3998	

0.0805				
(>= .121058)	97.56%	31.82%	57.01%	1.4309
0.0767				
(>= .1254..)	97.56%	33.33%	57.94%	1.4634
0.0732				
(>= .1307..)	95.12%	33.33%	57.01%	1.4268
0.1463				
(>= .1311)	95.12%	34.85%	57.94%	1.4600
0.1400				
(>= .1327..)	95.12%	36.36%	58.88%	1.4948
0.1341				
(>= .1347..)	95.12%	37.88%	59.81%	1.5312
0.1288				
(>= .1349..)	95.12%	39.39%	60.75%	1.5695
0.1238				
(>= .1393..)	95.12%	40.91%	61.68%	1.6098
0.1192				
(>= .1398..)	95.12%	42.42%	62.62%	1.6521
0.1150				
(>= .1440..)	95.12%	43.94%	63.55%	1.6968
0.1110				
(>= .1540..)	95.12%	45.45%	64.49%	1.7439
0.1073				
(>= .1583..)	95.12%	46.97%	65.42%	1.7937
0.1039				
(>= .1584..)	92.68%	46.97%	64.49%	1.7477
0.1558				
(>= .1767..)	90.24%	46.97%	63.55%	1.7017
0.2077				
(>= .1870..)	90.24%	48.48%	64.49%	1.7518
0.2012				
(>= .1896..)	90.24%	50.00%	65.42%	1.8049
0.1951				
(>= .2163..)	90.24%	51.52%	66.36%	1.8613
0.1894				
(>= .2182..)	90.24%	53.03%	67.29%	1.9213
0.1840				
(>= .225005)	90.24%	54.55%	68.22%	1.9854
0.1789				
(>= .2343..)	90.24%	56.06%	69.16%	2.0538
0.1740				
(>= .2367..)	87.80%	56.06%	68.22%	1.9983
0.2175				
(>= .2408..)	87.80%	57.58%	69.16%	2.0697
0.2118				
(>= .2461..)	85.37%	57.58%	68.22%	2.0122
0.2542				
(>= .2479..)	85.37%	59.09%	69.16%	2.0867
0.2477				
(>= .2583..)	82.93%	59.09%	68.22%	2.0271

					0.2889
(>= .2648..)	82.93%	60.61%	69.16%	2.1051	0.2817
(>= .2702..)	80.49%	60.61%	68.22%	2.0432	0.3220
(>= .2712..)	80.49%	62.12%	69.16%	2.1249	0.3141
(>= .2798..)	80.49%	63.64%	70.09%	2.2134	0.3066
(>= .2975..)	80.49%	65.15%	71.03%	2.3096	0.2995
(>= .3097..)	80.49%	66.67%	71.96%	2.4146	0.2927
(>= .3317..)	80.49%	68.18%	72.90%	2.5296	0.2862
(>= .3327..)	78.05%	68.18%	71.96%	2.4530	0.3220
(>= .3351..)	78.05%	69.70%	72.90%	2.5756	0.3150
(>= .3465..)	78.05%	71.21%	73.83%	2.7112	0.3083
(>= .3467..)	78.05%	72.73%	74.77%	2.8618	0.3018
(>= .3544..)	78.05%	74.24%	75.70%	3.0301	0.2957
(>= .3630..)	75.61%	74.24%	74.77%	2.9354	0.3285
(>= .386696)	73.17%	74.24%	73.83%	2.8407	0.3614
(>= .3890..)	73.17%	77.27%	75.70%	3.2195	0.3472
(>= .396343)	73.17%	78.79%	76.64%	3.4495	0.3405
(>= .4177..)	73.17%	80.30%	77.57%	3.7148	0.3341
(>= .4286..)	70.73%	80.30%	76.64%	3.5910	0.3645
(>= .4295..)	70.73%	81.82%	77.57%	3.8902	0.3577
(>= .4538..)	70.73%	83.33%	78.50%	4.2439	0.3512
(>= .4729..)	70.73%	84.85%	79.44%	4.6683	0.3449
(>= .4819..)	68.29%	84.85%	78.50%	4.5073	0.3737
(>= .5083..)	68.29%	86.36%	79.44%	5.0081	0.3671
(>= .5110..)	68.29%	87.88%	80.37%	5.6341	0.3608
(>= .5177..)	68.29%	89.39%	81.31%	6.4390	

0.3547				
(>= .5233..)	65.85%	89.39%	80.37%	6.2091
0.3820				
(>= .530951)	63.41%	89.39%	79.44%	5.9791
0.4093				
(>= .5322..)	63.41%	90.91%	80.37%	6.9756
0.4024				
(>= .540274)	63.41%	92.42%	81.31%	8.3707
0.3958				
(>= .5645..)	60.98%	92.42%	80.37%	8.0488
0.4222				
(>= .5951..)	58.54%	92.42%	79.44%	7.7268
0.4486				
(>= .6009..)	56.10%	92.42%	78.50%	7.4049
0.4750				
(>= .6486..)	56.10%	93.94%	79.44%	9.2561
0.4673				
(>= .653927)	53.66%	93.94%	78.50%	8.8537
0.4933				
(>= .6558..)	53.66%	95.45%	79.44%	11.8049
0.4855				
(>= .6602..)	51.22%	95.45%	78.50%	11.2683
0.5110				
(>= .6898..)	48.78%	95.45%	77.57%	10.7317
0.5366				
(>= .6998..)	46.34%	95.45%	76.64%	10.1951
0.5621				
(>= .717282)	46.34%	96.97%	77.57%	15.2927
0.5534				
(>= .7219..)	43.90%	96.97%	76.64%	14.4878
0.5785				
(>= .7394..)	43.90%	98.48%	77.57%	28.9756
0.5696				
(>= .7629..)	41.46%	98.48%	76.64%	27.3658
0.5944				
(>= .7723..)	39.02%	98.48%	75.70%	25.7561
0.6191				
(>= .7819..)	36.59%	98.48%	74.77%	24.1463
0.6439				
(>= .7842..)	34.15%	98.48%	73.83%	22.5366
0.6687				
(>= .7922..)	31.71%	98.48%	72.90%	20.9268
0.6934				
(>= .8020..)	29.27%	98.48%	71.96%	19.3171
0.7182				
(>= .8067..)	26.83%	98.48%	71.03%	17.7073
0.7430				
(>= .8083..)	24.39%	98.48%	70.09%	16.0976
0.7677				
(>= .8296..)	21.95%	98.48%	69.16%	14.4878

					0.7925
(>= .8496..)	19.51%	98.48%	68.22%	12.8780	0.8173
(>= .8580..)	17.07%	98.48%	67.29%	11.2683	0.8420
(>= .8613..)	14.63%	98.48%	66.36%	9.6585	0.8668
(>= .9014..)	14.63%	100.00%	67.29%		0.8537
(>= .9237..)	12.20%	100.00%	66.36%		0.8780
(>= .9331..)	9.76%	100.00%	65.42%		0.9024
(>= .93985)	7.32%	100.00%	64.49%		0.9268
(>= .9547..)	4.88%	100.00%	63.55%		0.9512
(>= .9766..)	2.44%	100.00%	62.62%		0.9756
(> .9766..)	0.00%	100.00%	61.68%		1.0000

Obs	ROC area	Std. err.	Asymptotic normal [95% conf. interval]	
107	0.8444	0.0402	0.76555	0.92329
