# Supplementary material for: Predictive model for aminoglycoside induced ototoxicity
Source: Front Neurol. 2024 Nov 1;15:1461823. doi: 10.3389/fneur.2024.1461823 (PMC11563990; doi:10.3389/fneur.2024.1461823)
Supplement: Supplementary file 2 [file Data_Sheet_2.pdf]

Detailed report of sensitivity and specificity

Cutpoint	Sensitivity	Specificity	Correctly classified	LR+	LR-
(>= .0228..)	100.00%	0.00%	26.09%	1.0000	
(>= .0395..)	100.00%	2.94%	28.26%	1.0303	0.0000
(>= .0410..)	100.00%	5.88%	30.43%	1.0625	0.0000
(>= .0701..)	100.00%	8.82%	32.61%	1.0968	0.0000
(>= .0758..)	100.00%	11.76%	34.78%	1.1333	0.0000
(>= .0958..)	100.00%	14.71%	36.96%	1.1724	0.0000
(>= .1032..)	91.67%	14.71%	34.78%	1.0747	0.5667
(>= .1064..)	91.67%	17.65%	36.96%	1.1131	0.4722
(>= .1070..)	91.67%	20.59%	39.13%	1.1543	0.4048
(>= .1288..)	91.67%	23.53%	41.30%	1.1987	0.3542
(>= .1306..)	91.67%	26.47%	43.48%	1.2467	0.3148
(>= .1523..)	91.67%	29.41%	45.65%	1.2986	0.2833
(>= .1889..)	91.67%	32.35%	47.83%	1.3551	0.2576
(>= .1920..)	91.67%	35.29%	50.00%	1.4167	0.2361
(>= .1950..)	83.33%	35.29%	47.83%	1.2879	0.4722
(>= .195956)	83.33%	38.24%	50.00%	1.3492	0.4359
(>= .226223)	83.33%	41.18%	52.17%	1.4167	0.4048
(>= .2468..)	83.33%	44.12%	54.35%	1.4912	0.3778
(>= .2576..)	83.33%	47.06%	56.52%	1.5741	0.3542
(>= .2694..)	83.33%	50.00%	58.70%	1.6667	0.3333
(>= .2714..)	83.33%	52.94%	60.87%	1.7708	0.3148
(>= .2833..)	83.33%	55.88%	63.04%	1.8889	

```
0.2982
( >= .3385.. )      83.33%          58.82%          65.22%          2.0238
0.2833
( >= .3613.. )      75.00%          58.82%          63.04%          1.8214
0.4250
( >= .3707.. )      75.00%          61.76%          65.22%          1.9615
0.4048
( >= .373711 )      75.00%          64.71%          67.39%          2.1250
0.3864
( >= .4194.. )      75.00%          67.65%          69.57%          2.3182
0.3696
( >= .4588.. )      75.00%          70.59%          71.74%          2.5500
0.3542
( >= .4867.. )      75.00%          73.53%          73.91%          2.8333
0.3400
( >= .4922.. )      66.67%          73.53%          71.74%          2.5185
0.4533
( >= .5715.. )      66.67%          76.47%          73.91%          2.8333
0.4359
( >= .5978.. )      58.33%          76.47%          71.74%          2.4792
0.5449
( >= .5984.. )      58.33%          79.41%          73.91%          2.8333
0.5247
( >= .6139.. )      58.33%          82.35%          76.09%          3.3056
0.5060
( >= .6474.. )      58.33%          85.29%          78.26%          3.9667
0.4885
( >= .7071.. )      58.33%          88.24%          80.43%          4.9583
0.4722
( >= .7289.. )      58.33%          91.18%          82.61%          6.6111
0.4570
( >= .7329.. )      58.33%          94.12%          84.78%          9.9167
0.4427
( >= .7528.. )      58.33%          97.06%          86.96%         19.8333
0.4293
( >= .7700.. )      50.00%          97.06%          84.78%         17.0000
0.5152
( >= .8091.. )      41.67%          97.06%          82.61%         14.1667
0.6010
( >= .8521.. )      33.33%          97.06%          80.43%         11.3333
0.6869
( >= .9001.. )      33.33%         100.00%          82.61%
0.6667
( >= .9356.. )      25.00%         100.00%          80.43%
0.7500
( >= .9519.. )      16.67%         100.00%          78.26%
0.8333
( >= .9888.. )       8.33%         100.00%          76.09%
0.9167
( >  .9888.. )       0.00%         100.00%          73.91%
```

1.0000

Obs	ROC area	Std. err.	Asymptotic normal [95% conf. interval]	
46	0.7917	0.0882	0.61885	0.96449
